# Naltrexone-Induced Cardiac Function Improvement is Associated With an Attenuated Inflammatory Response and Lipid Perioxidation in Volume Overloaded Rats

**DOI:** 10.3389/fphar.2022.873169

**Published:** 2022-06-30

**Authors:** Lukas Dehe, Shaaban A. Mousa, Mohammed Shaqura, Mehdi Shakibaei, Michael Schäfer, Sascha Treskatsch

**Affiliations:** ^1^ Department of Anesthesiology and Operative Intensive Care Medicine, Charité—Universitätsmedizin Berlin, Corporate Member of Freie Universität Berlin, Humboldt-Universität Zu Berlin and Berlin Institute of Health, Berlin, Germany; ^2^ Institute of Anatomy, Ludwig-Maximilians-Universität München, München, Germany

**Keywords:** naltrexone, cardiac, cytokine, lipid peroxidation, volume overload

## Abstract

In previous studies, upregulation of myocardial opioid receptors as well as the precursors of their endogenous ligands were detected in the failing heart due to chronic volume overload. Moreover, opioid receptor blockade by naltrexone improved left ventricular function. In parallel, inflammatory processes through cytokines have been confirmed to play an important role in the pathogenesis of different forms of heart failure. Thus, the present study examined the systemic and myocardial inflammatory response to chronic volume overload and its modulation by chronic naltrexone therapy. Chronic volume overload was induced in male Wistar rats by applying an infrarenal aortocaval fistula (ACF) for 28 days during which the selective opioid receptor antagonist naltrexone (*n* = 6) or vehicle (*n* = 6) were administered *via* a subcutaneously implanted Alzet minipump. The ultrastructural, morphometric and hemodynamic characterization of ACF animals were performed using an intraventricular conductance catheter *in vivo* and electron microscopy *in vitro*. Co-localization of mu-, delta- and kappa-opioid receptor subtypes (MOR, DOR, and KOR respectively) with the voltage gated L-type Ca2+ channel (Cav1.2), the ryanodine receptor (RyR), and mitochondria in cardiomyocytes as well as IL-6, IL-12, TNF-alpha, and Malondialdehyde (MDA) were determined using double immunofluorescence confocal microscopy, RT-PCR and ELISA, respectively. In rat left ventricular myocardium, three opioid receptor subtypes MOR, DOR, and KOR colocalized with Cav1.2, RyR and mitochondria suggesting a modulatory role of the excitation-contraction coupling. In rats with ACF-induced volume overload, signs of heart failure and myocardial ultrastructural damage, chronic naltrexone therapy improved cardiac function and reversed the systemic and myocardial inflammatory cytokine expression as well as lipid peroxidation. In conclusion, antagonism of the cardiodepressive effects of the myocardial opioid system does not only improve left ventricular function but also blunts the inflammatory response and lipid peroxidation.

## Introduction

Heart failure is a clinical syndrome characterized by inadequate cardiac function to maintain organ perfusion (McDonagh et al., 2021; Torregroza et al., 2021). The most common causes of heart failure are the acute events of myocardial ischemia and subsequent scaring or of chronic hypertension (Goel et al., 2013; De Luca, 2020). It is well known that heart failure is associated with significant morbidity and mortality worldwide (McDonagh et al., 2021). In contrast to the well described immediate cardioprotective effects of opioids in the acute event of myocardial ischemia (Torregroza et al., 2020; Torregroza et al., 2021), recent findings raised the awareness that the chronically enhanced intrinsic tone of endogenous opioids during heart failure may have detrimental effects (Treskatsch et al., 2015; Treskatsch et al., 2016; Dehe et al., 2021). Consistently, chronic morphine treatment resulted in a higher mortality risk in patients with acute coronary syndrome (Meine et al., 2005; Ray et al., 2016; Häuser et al., 2020) as well as in patients with chronic treatment of noncancer pain patients with long-acting opioids (Ray et al., 2016; Häuser et al., 2020). Indeed, intravenous morphine administration to elderly patients (age>75 years) suffering from congestive heart failure (CHF) increased their in-hospital mortality rate. Moreover, patients being administered morphine were more likely to develop congested heart failure (Ray et al., 2016; Häuser et al., 2020). In the ACF animal model of heart failure, the expression of myocardial opioid receptors as well as their endogenous ligands were highly up-regulated as part of a compensatory counter-regulation of the activation of the sympathetic nervous system (Treskatsch et al., 2014). In this context, persistent opioid receptor blockade by naltrexone significantly improved left ventricular function and also decreased rBNP-45 plasma concentrations in rats with volume overload (Dehe et al., 2021).

A growing body of evidence showed that neurohormones and cytokines, including IL-6 and TNF-alpha, contribute to the progression of heart failure (Seta et al., 1996). In more recent studies, inflammatory processes have been confirmed to be involved in the pathogenesis of different forms of CHF (Westermann et al., 2011; Libby et al., 2016). It is well established that particularly TNF-alpha is one of most crucial inflammatory cytokines which plays an important role in the pathogenesis and progression of heart disease (Feldman et al., 2000; Böse et al., 2007; Kleinbongard et al., 2010). Indeed, TNF-alpha is involved in multiple cellular progressions such as oxidative stress (Ozsoy et al., 2008) and apoptosis (Bryant et al., 1998; Condorelli, et al., 2002; Chen et al., 2014). Oxidative stress is known to impair mitochondrial function. In addition, a previous study demonstrated a correlation between increased mitochondrial oxidative stress and the rate of transcription of pro-inflammatory cytokines such as IL-6 in chronic human diseases (Naik and Dixit, 2011).

Therefore, there is great interest towards the elucidation of the potential therapeutic strategies targeting elevated cytokines and oxidative stress during heart failure. Since persistent opioid receptor blockade by naltrexone led to improved LV function and decreased rBNP-45 plasma concentrations in volume overloaded rats, the present study investigated whether cytokines such as IL-6, IL-12 and TNF-alpha as well as Malondialdehyde (MDA) plasma concentrations and their left ventricle myocardial content are significantly up-regulated during volume overload and whether the opioid receptor blockade by naltrexone resulted in the attenuation of this inflammatory response and lipid peroxidation.

## Materials and Methods

### Animals

Following approval by the local animal care committee (Landesamt für Gesundheit und Soziales, Berlin, Germany), we performed the present experiments in male Wistar rats (400 g) (Harlan Winkelmann, Borchen, Germany) according to the European Directive introducing new animal welfare and care guidelines (2010/63/EU). During the experimental period, rats were supported with standard laboratory chow and water *ad libitum* on a 12-h/12-h light–dark cycle.

### Aortocaval Fistula Induction and Naltrexone Treatment

The needle-technique to induce an infrarenal aortocaval fistula (ACF) has been described first by Garcia and Diebold using a 18 G needle (Garcia and Diebold, 1990). Here, we used an infrarenal aortocaval fistula (ACF) to induce chronic volume overload according to previous studies (Treskatsch et al., 2014). Briefly, under isoflurane anesthesia laparotomy was conducted and the aorta was penetrated with a 16 G disposable needle (Braun, Melsungen, Germany) distal to the renal arteries. Then, the needle was advanced across the aortic wall into the adjacent vena cava inferior. The aortic puncture site was sealed with cyanoacrylate glue after needle withdrawal needle. ACF patency was assessed by the pulsatile flow of oxygenated blood from the aorta into the vena cava inferior. Immediately at the end of ACF induction, group of 6 rats received a continuous subcutaneous administration of naltrexone (10 mg × kg^−1^ × h^−1^) by subcutaneously implanted Alzet^®^ minipumps (osmotic pump, model 2ML4, 2.5 μL per hour) (“ACF/Naltrexone”) (Dehe et al., 2021). Naltrexone binds with approximately similar affinity to three opioid receptor subtypes MOR, DOR and KOR (Ananthan et al., 1999). Naltrexone, compared with naloxone, exhibits a longer-acting opioid receptor antagonist effect, with a half-life of 3.9–10.3 h vs. approximately 60 min. The other group of rats (*n* = 5) with ACF also received a minipump delivering vehicle (isotonic saline) at the same volume during the whole experimental period (“ACF/Vehicle”). Sham-operated animals (*n* = 6) served as controls subjected to the same operational steps except the puncture of the aorta and without any treatment. The subcutaneously injected metamizole (40 mg/kg) was used as post-surgical analgesia (Treskatsch et al., 2014).

### Hemodynamic Evaluation

The “closed chest” method was used to measure hemodynamic parameters as previously described (Treskatsch et al., 2014). Briefly, 28 days after fistula induction in the measurements were done under tiletamine/zolazepam anesthesia (Zoletil^®^, 10 mg/kg s.c. followed by 50 mg/kg i.m.) in spontaneously breathing rats (Saha et al., 2007). Following anesthesia (5–10 min), rats subjected to tracheostomy to facilitate spontaneous breathing and were placed on a heating pad to maintain body temperature. In order to measure the central venous pressure (CVP) a plastic catheter (PE-50) was inserted via the left jugular vein into the superior vena cava. However, the arterial and intraventricular pressures and their derivatives were determined with a pressure micro-tip catheter (Millar®, SPR-838 NR), which was inserted into the left ventricle via the right carotid artery. All parameters were recorded and analyzed by the PowerLab®-system and software (AD Instruments, Dunedin, New Zealand). At the end of experiments, rats were decapitated and their hearts were removed by dissecting the aortic root immediately above the aortic valves and the superior vena cava above the atria; then the entire heart weight was determined. Experiments were performed in triplicate at 10 min intervals to assure stable measurement conditions.

### Immunofluorescence and Electron Microscopy

Control or rats with ACF-induced volume overload were deeply anesthetized with tiletamine/zolazepam (Zoletil®) and transcardially perfused with 100 ml warm saline, followed by 300 ml 4% (w/v) paraformaldehyde in 0.16 M phosphate buffer solution (pH 7.4) (“fixative solution”). Then, left ventricle were removed, postfixed in fixative solution, and cryoprotected overnight at 4°C in PBS containing 10% sucrose. The 10 μm thick sections of the left ventricle were mounted onto gelatin-coated slides and incubated overnight with the following primary antibodies as described previously (Treskatsch et al., 2014; Treskatsch et al., 2015; Treskatsch et al., 2016) as follows: rabbit polyclonal anti-Mu-Opioid Receptor (MOR) (1:1000) (gift from S. Schulz and V. Höllt, Magdeburg, Germany), rabbit polyclonal anti-Delta-Opioid Receptor (DOR) (Dr. R. Elde, Minneapolis, MN, United States), rabbit polyclonal anti-Kappa-Opioid Receptor (KOR) (1:1000) (gift from S. J. Watson, Michigan, United States) in combination with the mouse monoclonal anti-dihydropyridine receptor (ɑ2 subunit) antibody to identify the voltage-gated L-type Ca2+ channel (anti-Cav1.2) (SIGMA^®^, Missouri, United States), monoclonal anti-Inositol-1,4,5-trisphosphate receptor type III (1:600) to identify ryanodine receptors (RyR) (BD Biosciences) or monoclonal anti-mitochondrium marker MTC02 (1:300) to identify mitochondrial structures (Thermo Scientific). After incubation, with primary antibodies, the tissue sections were then washed with PBS and incubated with Texas Red-conjugated goat anti-rabbit antibody (Vector Laboratories) and FITC-conjugated donkey anti-mouse secondary antibodies (Vector Laboratories, Inc. Burlingame, CA). Thereafter, sections were washed with PBS and the nuclei stained bright blue with 4′-6-diamidino-2-phenylindole (DAPI) (0.1 μg/ml in PBS) (SIGMA^®^, Missouri, United States). Finally, the tissues were washed in PBS, mounted on Vectashield (Vector Laboratories), and viewed under a Zeiss LSM 510 laser scanning microscope (Carl Zeiss, Göttingen, Germany). To demonstrate specificity of staining, the following controls were included: omission of the primary antisera or the secondary antibodies, as described in previous studies (Ananthan et al., 1999; Naik and Dixit, 2011; Treskatsch et al., 2014). For electron microscopy evaluation as described previously (Treskatsch et al., 2015), samples were fixed in Karnovsky’s fixative and then post-fixed in 2% OsO4/0.1 M phosphate buffer. After rinsing and dehydration in ethanol, the samples were embedded in Epon (Plano, Marburg, FRG), ultrathin cuts made on a Reichert Ultracut E, and contrasted with 2% uranyl acetate and lead citrate. A transmission electron microscope (Zeiss TEM10, Jena, Germany) was used to examine the tissue samples.

### Quantification of Immunostaining

Images were obtained on a confocal laser scanning microscope, LSM510, equipped with an argon laser (458/488/514 nm), a green helium/neon laser (543 nm), and a red helium/neon laser (633 nm; Carl Zeiss, Göttingen, Germany). Single optical slice images were taken using ×10 or ×20 Plan-Neofluar air interface or ×40 Plan-Neofluar oil interface objective lens. The settings of the confocal microscope were established using a control section and kept unchanged for all subsequent acquisitions. For the quantitative evaluation of all immunofluorescence double staining of opioid receptor subtypes with Cav1.2 or RyR, the version 1.41 of the image analysis program ImageJ® was applied (http://rsbweb.nih.gov/ij/) as previously described (Shimojo et al., 1997; Schneider et al., 2012). Briefly, the different color channels, each identifying distinct target structures, were separated by using the plug-in (color deconvolution), thus the color signal can quantitatively be evaluated. A manually specified area was identified for each specifically colored area. Intensity thresholds were assigned, so that a maximum degree of integrated area of stained target structure was identified, while minimizing possible background activities. Areas above the threshold value were defined as positive and indicated information about the percentage of the immunostained area in relation to the previously selected total area. Values below the threshold were eliminated as background. The threshold value was kept constant for all sections. With the help of ImageJ, the parameter percentage area (% stained area) was calculated using the software. The percentage area was defined as the specific-colored area in relation to the total area of a photographed tissue preparation. All calculated quantitative color intensities are presented as % immunoreactive area in the manuscript (see also Supplementary Figure S1). Data were presented as median plus range.

### Determination of Inflammatory Cytokines and Lipid Peroxidation

Blood samples from control (*n* = 6), ACF/Vehicle (*n* = 5) and ACF/Naltrexone (*n* = 6) animals were withdrawn into EDTA-preloaded tubes after completion of hemodynamic measurements in order to measure the serum inflammatory cytokines concentrations and lipid peroxidation. Then, the blood was centrifuged at 1,000 g for 10 min at 4°C immediately after withdrawal. Subsequently, the plasma was maintained at −80°C until further use. Finally, a sensitive enzyme-linked immunosorbent assay (ELISA) kit (Abnova, Heidelberg, Germany) was used to measure the plasma concentrations of IL-6, IL-12, TNF-alpha and Malondialdehyde (MDA).

### Quantitative RT-PCR of Myocardial Inflammatory Cytokine mRNA

PCR analysis for IL-6, IL-12, TNF-alpha and Malondialdehyde specific mRNA from rat left ventricle myocardium was performed by using the commercially available Qiazol Lysis kit, (Qiagen, Hilden, Germany) as described previously (Dehe et al., 2021). Total RNA was extracted from the entire left ventricle myocardium of Wistar rats (*n* = 6 per experimental group) using RNeasy Kit (Qiagen, Hilden, Germany). 0.5 µL (25 pmol) oligo dT and 2 µL (200 pmol) random primers were added up to 1 μg total RNA, incubated at 37°C for 15 min, then at 85°C for 5 s, finally at 4°C for transfer onto ice (according to TaKaRa® manual). cDNA was stored at −20 C. Taqman® qRT-PCR was performed with a SYBR® Green kit following the manufacturer’s instructions (Applied Biosystems). The following specific primers were used: for IL-12, forward primer: GCA​TGT​GTC​AAT​CAC​GCT​ACC, reverse primer: AAG​ACA​CTT​GGC​AGG​TCC​AG (Ensembl, Accession Nr: NM_053390.1); for IL-6, forward primer: GTTTCTCTCCGCAA GAGACTT, reverse primer: TGG​TCT​GTT​GTG​GGT​GGT​ATC (Ensembl, Accession NM_012589); for TNF-alpha, forward primer: GTGATCGGTCCCAA CAAGGA, reverse primer: CGC​TTG​GTG​GTT​TGC​TAC​G (Ensembl, Accession Nr: NM_012675.3). Amplification was carried out for 40 cycles, each consisting of 15 s at 95°C; for cytokines specific mRNA and 18S ribosomal protein for 60 s at 60°C. A temperature just below the specific melting temperature (Tm) was employed for detection of fluorescence specific products. IL-12, IL-6 and TNF-alpha specific mRNA were quantified using three independent samples in duplicate. The housekeeping gene S18, a ribosomal protein, was used as an internal reference gene for quantification.

### Statistical Analyses

The acquired data were expressed as medians plus their interquartile ranges. Statistical differences between the three groups were obtained using a one-way analysis of variance (ANOVA) on Ranks (Kruskal–Wallis test) followed by post hoc Dunn’s test. Sigma Plot 13.0 statistical software (Systat Software GmbH, Erkrath, Germany) was used to perform all the statistical test.

## Results

### Localization of Mu-Opioid Receptor, Delta-Opioid Receptor, and Kappa-Opioid Receptor in the Left Ventricle Myocardium

**FIGURE 1:**
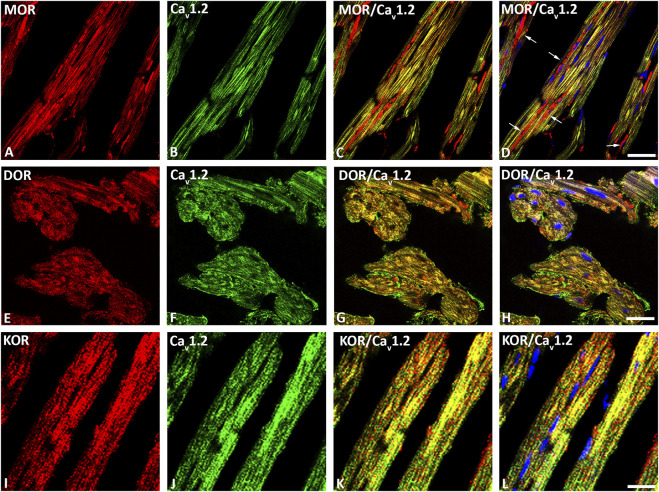
Double immunofluorescence confocal microscopy of opioid receptor mu (MOR) **(A)**, delta (DOR) **(E)**, and kappa (KOR) **(I)** (Texas red immunofluorescence) with L-type Ca^2+^-channel Cav1.2 (FITC green fluorescence) **(B**, **F**, **J)** in rat left ventricular myocardium. Note that rows of mitochondria are located between cardiomyocytes and are immunostained for MOR as indicated by the red fluorescence (arrow) **(D)**. Nuclei identified by their bright blue using DAPI staining **(D**, **H**, **L)**. **(C**–**L)** show the colocalization of opioid receptor subtypes mu, delta and kappa together with the L-type Ca^2+^-channel Cav1.2 on cardiomomyocytes of the left ventricle. Bar = 20 μm.

**FIGURE 2:**
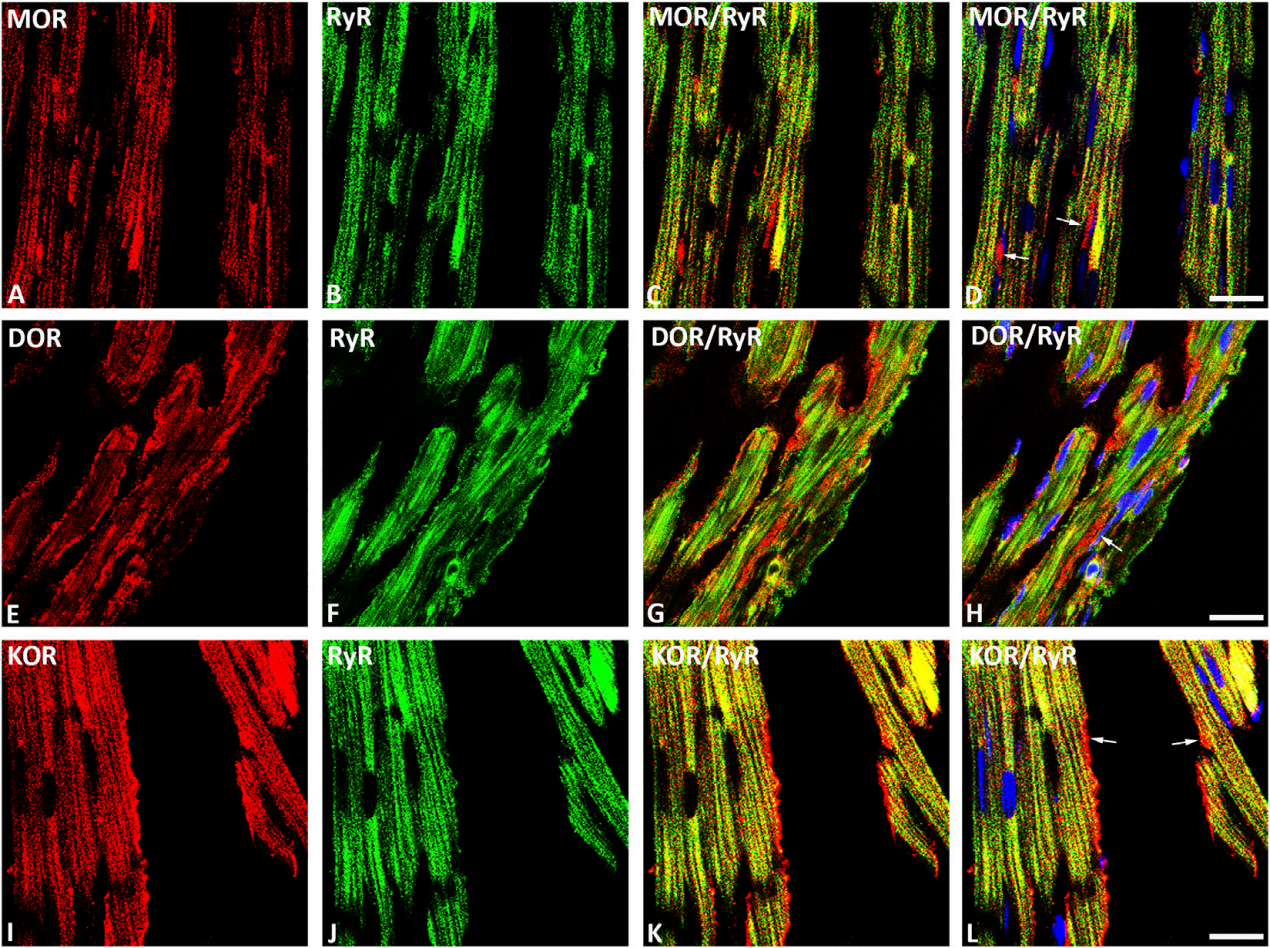
Double immunofluorescence confocal microscopy of opioid receptor subtypes mu (MOR) **(A)**, delta (DOR) **(E)**, and kappa (KOR) **(I)** (Texas red immunofluorescence) in combination with the sarcoplasmatic ryanodine receptor (RyR) (FITC green fluorescence) **(B**, **F**, **J)** in rat left ventricular myocardium. **(C**–**L)** show co-expression of opioid receptor subtypes MOR, DOR and KOR with RyR on the cardiomyocytes of the rat left ventricle (yellow fluorescence). Note that rows of mitochondria are located between cardiomyocytes and are immunostained for MOR **(D)**, DOR **(H)** or KOR **(L)** as indicated by the red fluorescence (arrow). Nuclei are indicated by their bright blue fluorescence **(D**, **H**, **L)**. Bar = 10 μm.

Our double immunofluorescence confocal microscopy showed in tissue sections of rat left ventricular myocardium the presence of the opioid receptor subtypes MOR, DOR and KOR, their colocalization with the voltage-gated L-type Ca2+-channel Cav1.2 on the outer cell membrane (Figure 1) and their colocalization with the ryanodine receptor (RyR) on the intracellular sarcoplasmic reticulum of left ventricular cardiomyocytes (Figure 2). Quantification of the immunohistochemical staining of these images by ImageJ (Version 1.52a, NIH, United States) imaging software provided the median [range]% values of the area of Cav1.2-immunoreactivity colocalizing with distinct opioid receptor subtype immunoreactivity (yellow fluorescence) of up to 56 (48–65)% for MOR, 67 (55–80)% for DOR and 78 (75–91)% for KOR. Moreover, quantification of the median (range)% values of the area of RyR-immunoreactivity colocalizing with distinct opioid receptor subtype immunoreactivity revealed an overlap (yellow fluorescence) of up to 59 (51–94)% for MOR, 77 (66–84)% for DOR and 63 (60–73)% for KOR (Figures 2A–L).

**FIGURE 3:**
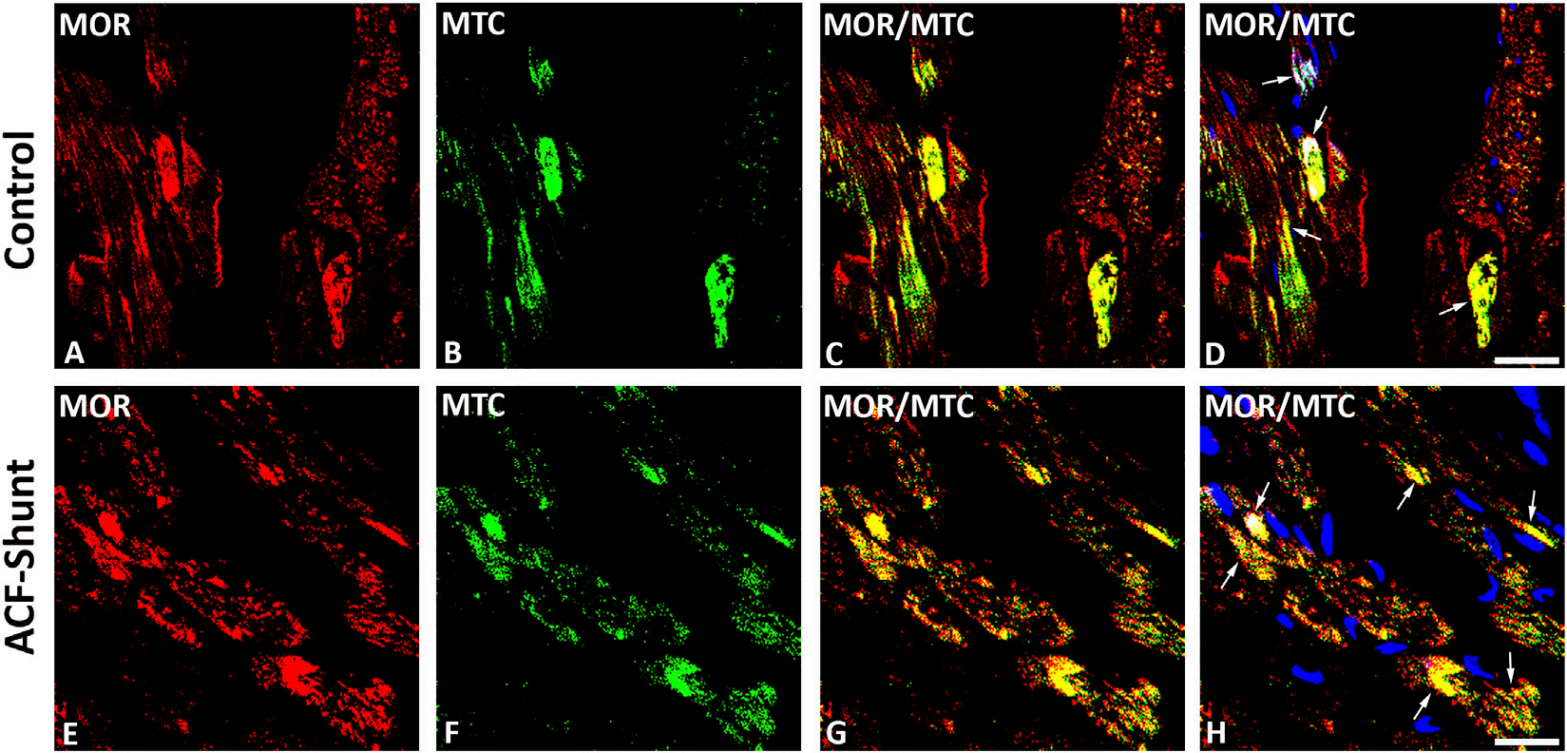
Double immunofluorescence staining of mu-opioid receptor (MOR) **(A**, **E)** (red fluorescence) and mitochondrial marker MCT02 (green fluorescence) **(B**, **F)** in rat left ventricular myocardium from controls **(A**–**D)** and rats with ACF-induced volume overload **(E**–**H)**. **(A**–**D)** show that some of MOR immunoreactivity colocalized intracellularly with well-defined mitochondrial structures (mitochondrial marker MCT02) (colocalization seen as yellow immunofluorescence) (arrow). **(E**–**H)** show MOR colocalized with swollen mitochondrial structures (mitochondrial marker MTC02) of rat LV cardiomyocytes. (Bar = 10 µm).

MOR, DOR and KOR single staining (Texas red fluorescence) were consistently demonstrated in mitochondria-like structures which were regularly arranged in rows located between cardiomyocytes (Figures 1, 2). Consistently, MOR (but also DOR and KOR, data not shown) colocalized with well-defined mitochondrial structures (mitochondrial marker MTC02) of left ventricular cardiomyocytes from controls (Figures 3A–D) compared to ACF-induced volume overload rats (Figures 3E–H).

### Cardiac Remodeling in Aortocaval Fistula Rats

28 days after fistula induction, the weight indices of heart (*p* = 0.008) as well as lung (*p* = 0.006) exhibited a significant increase in all rats with ACF-induced volume overload compared to controls (Table 1). However, chronic naltrexone treatment of ACF rats did not significantly affect heart and lung weight indices compared to the ACF/Vehicle treated group (Table 1).

**TABLE 1:** Changes of morphometric and hemodynamic parameters of the weight indices of heart and lung in relation to body weights and biventricular filling pressures obtained from control rats and vehicle- (isotonic saline) or naltrexone-treated rats with ACF-induced volume overload. The data are given as medians plus interquartile ranges: HR = heart rate; SBP = systolic blood pressure; DBP = diastolic blood pressure; BW = body weight. Note that there are significant differences not only between the ACF/Vehicle and control group (P1-value) but also between the ACF/Vehicle and ACF/Naltrexone group (P2-value). *p* < 0.05 was considered statistically significant.

	Control (*n* = 6)	ACF/Vehicle (*n* = 5)	ACF/Naltrexone (*n* = 6)	*p*-value
Body weight (g)	409 (331; 453)	415 (366; 468)	397 (356; 449)	P = 0.664
Heart weight (mg)	1550 (1425; 1900)	2225 (1790; 2825)	1942 (1625; 2410)	P_1_ = 0.003
				P_2_ = 0.559
Heart/BW (mg/g kg)	3.6 (3.5; 4.3)	5.7 (4.3; 5.8)	4.5 (3,2; 5.5)	P_1_ = 0.008
				P_2_ = 0.326
Lung/BW (mg/g kg)	3.9 (3.2; 4.5)	5.4 (4.9; 8.9)	5.3 (5.1; 6.2)	P_1_ = 0.006
				P_2_ = 1.00
SBP (mmHg)	155 (141; 179)	118 (106; 130)	142 (108; 176)	P_1_ = 0.016
				P_2_ = 0.213
DBP (mmHg)	130 (102; 153)	76 (65; 84)	94 (73; 108)	P_1_ = 0.003
				P_2_ = 0.743
LVEDP (mmHg)	4.9 (4.6; 5.6)	10.8 (8.3; 16.4)	6.8 (6.2; 7.7)	P_1_ = 0.005
				P_2_ = 0.021
dP/dt_max_ (mmHg/s)	17890 (14365; 18562)	9068 (6974; 10795)	14124 (12785; 15006)	P_1_ = 0.005
				P_2_ = 0.011
dP/dt_min_ (mmHg/s)	−10937 (−13344;−9058)	−6659 (−7530; −3614)	−8675 (−9764; −7813)	P_1_ = 0.002
				P_2_ = 0.026
rBNP-45 (pg/ml)	27 (17; 42)	141 (133; 171)	36 (32; 46)	P_1_ = 0.002
				P_2_ = 0.044
Angiotensin-2 (pg/ml)	386 (371; 456)	1092 (989; 1135)	430 (393; 473)	P_1_ = 0.001
				P_2_ = 0.059

**FIGURE 4:**
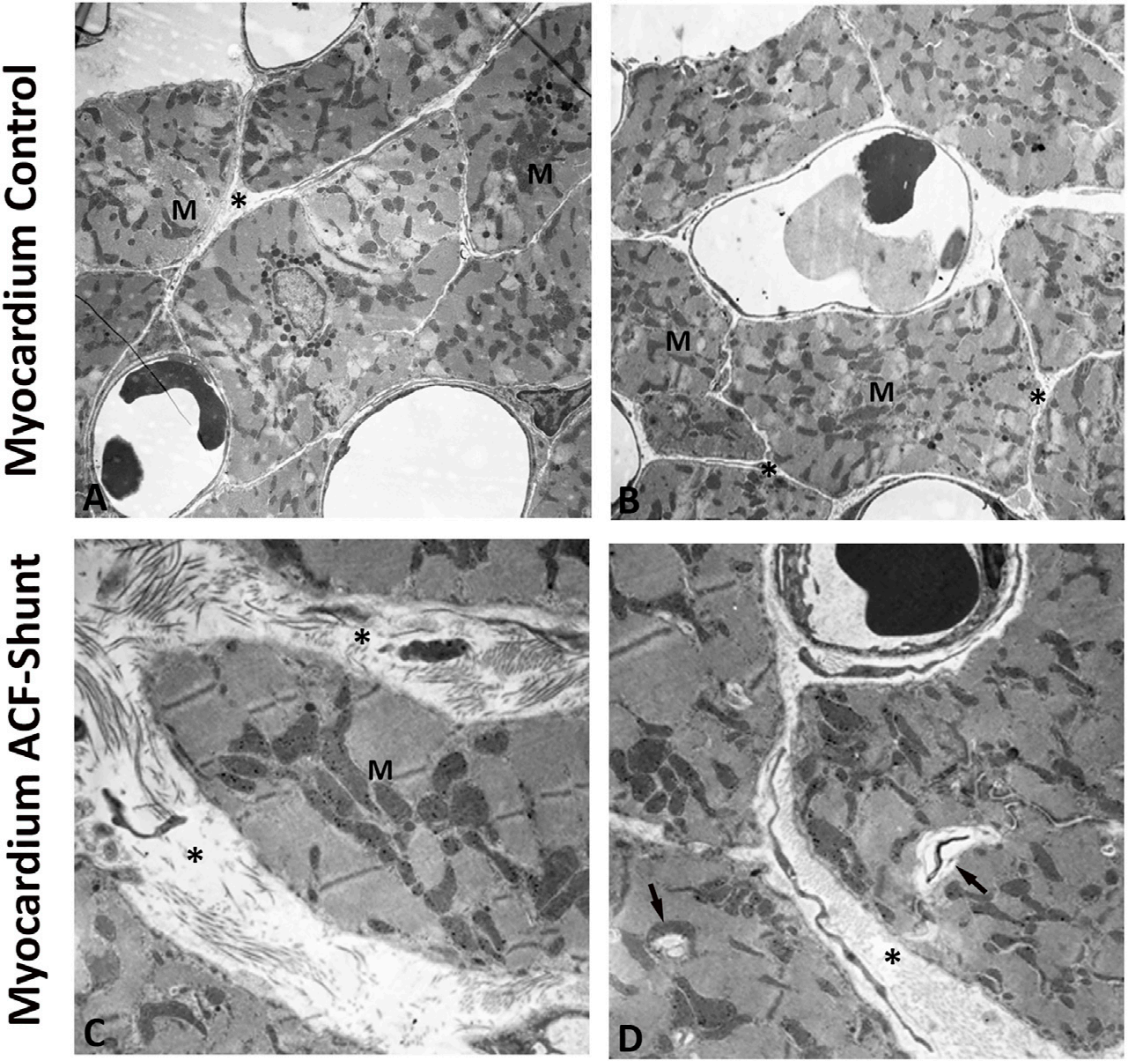
Electron micrographs of the left ventricle (LV) from controls **(A**, **B)** and rats with ACF-induced volume overload **(C**, **D)**. A and B display cross sections of control hearts showing intact myocardial tissue with specific cell–cell contacts and regular intercellular spaces (see *), cardiomyocytes with well-organized numerous cardiomyofibers and intact mitochondria with a preserved internal architecture. **(C**, **D)** show ultrastructure changes of the left ventricle at 28 ± 2 days after ACF induction. Note the widening of the intercellular space (see *), degenerated myofibers (arrow) and disorganized cell organelles. In addition, many mitochondria (M) showed ultrastructural abnormalities such as size enlargement with amorphous dense bodies and disruption of the internal architecture. Magnification: **(A)** × 10.000; **(B)** × 20.000, bar = 1 μm.

Ultrastructural analysis of the left ventricle myocardium in normal (control) rats revealed intact myocardial tissue with specific cell–cell contacts and regular intercellular spaces, cardiomyocytes with well-organized numerous cardiomyofibers and normal mitochondrial distribution with a preserved internal architecture (Figures 4A,B). In contrast, left ventricle myocardium of ACF rats showed widening of the intercellular space, disrupted contractile structures and cellular fragmentation. Moreover, the mitochondria exhibited a poorly preserved internal architecture including size enlargement and accumulation of amorphous dense bodies (Figures 4C,D).

### Naltrexone Improved Cardiac Function

In all rats with ACF-induced volume (*n* = 5) overload, there was a significant increase in biventricular filling pressures (LVEDP: *p* = 0.005) and significant decrease in the diastolic blood pressure (DPB: *p* = 0.003) compared to controls (*n* = 6) due to the aortocaval fistula (Table 1). Chronic naltrexone treatment of ACF rats (*n* = 6) caused a significant reduction in left ventricular end-diastolic pressure (LVEDP, *p* = 0.021) compared to ACF rats subjected to vehicle treatment (Table 1). Moreover, global myocardial contractility as measured by dP/dt_min_ was significantly improved due to chronic naltrexone treatment in ACF rats (dP/dt_min_: *p* = 0.002) (Table 1). Volume overload significantly increased rBNP-45 (*p* < 0.002) and angiotensin-2 (*p* < 0.001) plasma levels in ACF/vehicle rats and this increase was reversed by naltrexone treatment (Table 1).

### Attenuated Cytokine Response in Rats With Naltrexone Treatment

**FIGURE 5:**
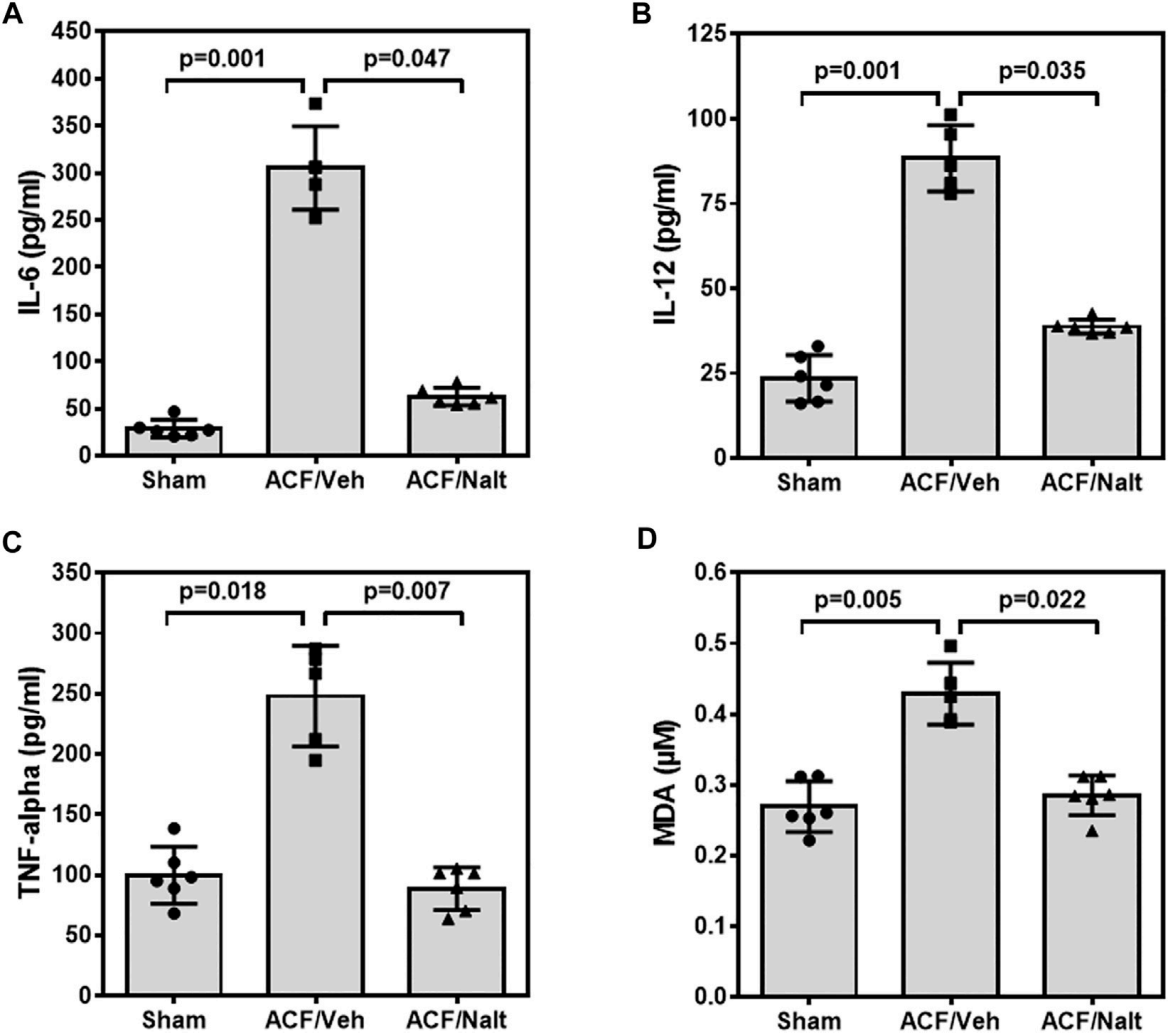
Interleukin 6 (IL-6) **(A)**, Interleukin 12 (IL-12) **(B)**, tumor necrosis factor α (TNF-α) **(C)** and malondialdehyde (MDA) **(D)** values of control, ACF/Vehicle- and ACF/Naltrexone-treated rats. Values of inflammatory cytokines and malondialdehyde were significantly increased in ACF/Vehicle treated rats compared to controls (IL-6: *p* = 0.001; IL-12: *p* = 0.001; TNF-alpha: *p* = 0.018; MDA: *p* = 0.005). Chronic naltrexone treatment in ACF rats was concomitant with a significant decrease in the inflammatory cytokines and lipid-peroxidation (IL-6: *p* = 0.047; IL-12: *p* = 0.035; TNF-alpha: *p* = 0.007; MDA: *p* = 0.022). Values are medians plus interquartile ranges (*n* = 6 rats/group). Data represent medians (range).

**FIGURE 6:**
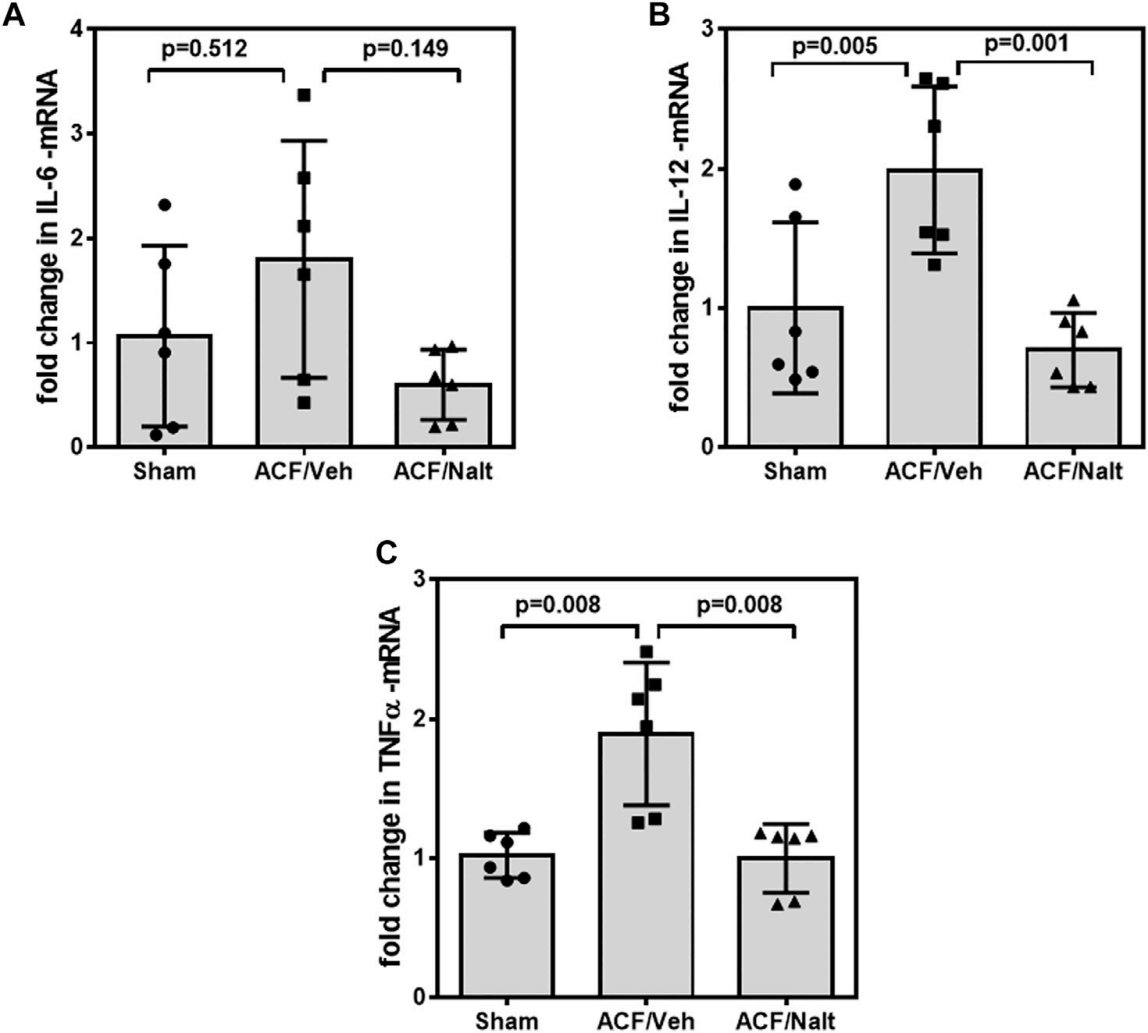
Quantitative RT-PCR Interleukin 6 (IL-6) **(A)**, Interleukin 12 (IL-12) **(B)** and tumor necrosis factor α (TNF-alpha) **(C)** specific mRNA values of control, ACF/Vehicle- and ACF/Naltrexone-treated rats. Values of inflammatory cytokines were significantly increased in ACF/Vehicle treated rats compared to controls (IL-12: *p* = 0.005; TNF-alpha: *p* = 0.008) but not for IL-6 (*p* = 0.512). Chronic naltrexone treatment in ACF rats was concomitant with a significant decrease in the inflammatory cytokine mRNA (IL-12: *p* = 0.001; TNF-alpha: *p* = 0.008) but not for IL-6 (*p* = 0.149). Data represent medians (range) (*n* = 6 rats/group).

Volume overload led to a significant increase of plasma inflammatory cytokines and lipid peroxidation in ACF/vehicle rats (*n* = 5) (IL-6: *p* = 0.001; IL-12: *p* = 0.001; TNF-alpha: *p* = 0.018; MDA: *p* = 0.005) (Figures 5A–D). However, chronic naltrexone treatment in ACF rats (*n* = 6) was associated with a significant reduction of the inflammatory cytokines including IL-6, IL-12 as well as TNF-alpha in the blood plasma (IL-6: *p* = 0.047, IL-12: *p* = 0.035, TNF-alpha: *p* = 0.007) (Figures 5A–D). Moreover, chronic naltrexone administration in ACF rats (*n* = 6) reduced lipid peroxidation as measured by malondialdehyde (MDA: *p* = 0.022) (Figures 5A–D). In parallel, volume overload led to a significant increase in the mRNA transcription of the inflammatory cytokines IL-12 and TNF-alpha within the myocardium of the left ventricle of ACF/vehicle rats (IL-12: *p* = 0.005, TNF-alpha: *p* = 0.008) but not for IL-6 (*p* = 0.512) (Figures 6A–C). Importantly, chronic naltrexone treatment in ACF rats was associated with a significant reduction of the inflammatory cytokine IL-12, and TNF-alpha mRNA (IL-12: *p* = 0.001, TNF-alpha: *p* = 0.008) but not for IL-6 (*p* = 0.149) (Figures 6A–C).

## Discussion

This study confirms the co-expression of opioid receptor subtypes MOR, DOR and KOR with the voltage-gated L-type Ca2+-channels Cav1.2 and the intracellular ryanodine receptor in rat LV cardiomyocytes. Moreover, opioid receptor subtypes also colocalized with mitochondria of cardiomyocytes. In rats with ACF-induced volume overload, hemodynamic signs of heart failure were associated with myocardial ultrastructural damage, an enhanced systemic (increased IL-6, IL-12, TNF-a plasma concentrations) as well as myocardial (increased IL-6, IL-12, TNF-a mRNA transcription) inflammatory response. In parallel, the mitochondrial lipid peroxidation was enhanced. Interestingly, these harmful effects were reversed by chronic naltrexone treatment in rats with ACF-induced volume overload. Taken together, antagonism of the cardio-depressive effects of the myocardial opioid system does not only improve left ventricular function but also blunts the inflammatory response and lipid peroxidation.

Similar to the demonstrated colocalization of opioid receptors with voltage gated calcium channels in neurons of the central nervous system (Takasusuki and Yaksh, 2011) and the localization of G protein coupled receptors such as the adrenergic and opioid receptors in both the sarcolemma and intracellular microdomains of cardiomyocytes (Head et al., 2005), the present study shows abundant colocalization of opioid receptor subtypes with the calcium channels Cav1.2 and RyR as well as the mitochondria of rat LV cardiomyocytes. Consistently, recent study by Weis and Zamponi (Weiss and Zamponi, 2021) demonstrated the inhibitory effects of opioids on calcium channels. Collectively, these findings seem to point towards a functional link between cardiac opioid receptors and the excitation–contraction coupling within cardiomyocytes.

In animals with volume overload–due to an aorto-caval shunt–our study confirms previous findings (Garcia and Diebold, 1990; Brower et al., 1996; Dehe et al., 2021) that the significant increase of the weight indices of both the heart and lung in rats with ACF-induced volume overload was associated with a marked increase in the rBNP-45 and angiotensin-2 plasma concentrations. In parallel, there was a significant change in all hemodynamic measurements of ACF rats indicating the pronounced systolic and diastolic left ventricular dysfunction. Indeed, our ACF model is characterized by severe biventricular dilatation with signs of decompensated heart failure as shown by a significantly reduced cardiac contractility and elevated LVEDP (Treskatsch et al., 2014). Consistently, our electron microscopy analysis showed clear ultrastructural damage of the LV myocardium in ACF rats with volume overload.

Today, neurohormones and cytokines are known to play an essential role in the development of heart failure (Seta et al., 1996; Van Linthout and Tschöpe, 2017). A growing body of evidence shows that neurohormones and cytokines, including IL-6 and TNF-alpha, contribute to the progression of heart failure (Seta et al., 1996). Indeed, our study showed that volume overload in ACF rats caused a significant increase in systemic plasma levels of inflammatory cytokines including IL-6, IL-12 and TNF-alpha as well as lipid-peroxidation concomitant with the enhanced expression of IL-6, IL-12 and TNF-alpha mRNA within myocardium. These findings are in in agreement with the notion that inflammatory processes have been recognized to be a cornerstone in the pathogenesis of different forms of CHF (Westermann et al., 2011; Libby et al., 2016) and that oxidative stress has been shown to be involved heart failure in animals and humans (Hokamaki et al., 2004; Polidori et al., 2004; LeLeiko et al., 2009). Consistent with these results, patients suffering from heart failure exhibited a correlation between enhanced expression of serum inflammatory cytokines and adverse clinical outcomes (Torre-Amione et al., 1996; Hokamaki et al., 2004). Indeed, TNF-α, IL-1ß and IL-6 levels were increased in CHF patients and TNF-α correlated with the severity of the disease (Torre-Amione et al., 1996). Moreover, these cytokines and their corresponding receptors were independent indicators of mortality rate in patients with progressed CHF (Deswal et al., 2001). In parallel, cytokine, including TNF-alpha, treatment reduced both contraction and the Ca2^+^ transient in rat ventricular myocytes and these effects were modulated by opioids (Duncan et al., 2007). Moreover, TNF-alpha seems to have negative inotropic effects due to the changes in intracellular Ca2+ homeostasis within adult cardiomyocytes (Yokoyama et al., 1993), most likely via downregulation of Ca2+-regulating genes (Thaik et al., 1995; Wu et al., 2011).

Interestingly the present experiments demonstrated that the improved cardiac function in rats with ACF-induced volume overload by chronic naltrexone treatment reversed the enhanced expression of inflammatory cytokines IL-6, IL-12 and TNF-alpha within myocardium as well as in serum. Moreover, naloxone inhibited endotoxin-induced up-regulation of inflammatory molecules including IL-6 and TNF-alpha as well as NF-kB activation through antagonizing the L-type calcium channels (Jan et al., 2011). In contrast, chronic administration of tramadol in normal rats enhanced the expression of serum inflammatory cytokines and apoptotic markers as well as lipid peroxidation in the cerebrum of rats (Mohamed and Mahmoud, 2019).

Cardiomyocytes energy requirement is covered by mitochondria to maintain their contractile function. In case of a higher energy demand, cardiomyocytes produce new mitochondria (mitochondrial biogenesis) (Ayoub et al., 2017). The malfunction of mitochondrial biogenesis occurs in heart failure in humans and animal models of pressure overload (Sebastiani et al., 2007; Witt et al., 2008). The restriction in heart function is the consequence of apoptosis and myocardial remodeling initiated by mitochondria. In addition, oxidative stress is defined as a state when reactive oxygen species (ROS) defeat the body’s antioxidant enzymes. ROS are oxygen-containing molecules that are chemically active and formed as a by-product of oxygen metabolism (Ayoub et al., 2017). In this study, the chronic blockade of the cardiac opioid system by naltrexone resulted in a reduced lipid peroxidation as a surrogate of oxidative stress. In this context, oxidative stress is known to impair mitochondrial function leading to a reduced mitochondrial capacity to generate ATP. Sharov showed this in dogs with chronic heart failure due to myocardial ischemia (Sharov et al., 1998). Extending the aforementioned studies, we showed the intracellular colocalization between opioid receptors and mitochondria in cardiomyocytes of the left ventricle in rats suggesting a modulatory role in mitochondrial function under conditions of oxidative stress.

Several limitations should be considered. Since our animal model does not represent the multimorbidity and causality of cardially compromised patients, one cannot transfer these finding directly to a clinical situation. However, this animal model has been extensively described and, within 28 days, the animals exhibited a predictable state of nearly decompensated heart failure accompanied by a dilatative cardiomyopathy. It is characterized by significantly elevated biventricular filling pressures and reduced cardiac contractility. The rats typically show overt signs of decompensation, e.g., ascites, strained breathing, decreased mobility, and sudden arrhythmia. The study was conducted only in male Wistar rats. Female Wistar rats are prone to a more difficult ACF induction, and standardization has not been established yet. Therefore, a comprehensive analysis of this experimental model amongst female Wistar rats has yet to be conducted.

## Conclusion

In summary, the present findings give evidence of the essential role of the intrinsic cardiac opioid system during chronic volume overload. Morphologically, opioid receptor subtypes were colocalized with calcium channels as well as mitochondria in cardiomyocytes of the LV in rats suggesting a modulatory role of the excitation-contraction coupling. This was accompanied by an increased expression of inflammatory cytokines and TNF-alpha as well as elevated lipid peroxidation in the blood circulation and in left ventricle myocardium. Importantly, chronic naltrexone treatment attenuated these signs of a systemic and local inflammatory response and lipid peroxidation in parallel to an improved LV function and reduced rBNP-45 plasma concentration. Since the naltrexone treatment did not completely reverse the measured parameters to baseline, it cannot be ruled out that there are also other mechanisms responsible.

## Data Availability

The original contributions presented in the study are included in the article/Supplementary Material, further inquiries can be directed to the corresponding authors.

## Supplementary Material

Supplementary Figure S1 Example of quantitative evaluation of immunohistochemical staining within rat atria using the version 1.41 of the image analysis program ImageJ^®^ (http://rsbweb.nih.gov/ij/). The additional use of the plug-in (color deconvolution) allowed the separation of the different color channels each identifying distinct target structures, whose color signal can, thus, be quantitatively evaluated. With the help of ImageJ, the parameter percentage area (% stained area) was calculated. The percentage area was defined as the specific-colored area in relation to the total area of a photographed tissue preparation.
