# Supplementary figures and images for: Naltrexone-Induced Cardiac Function Improvement is Associated With an Attenuated Inflammatory Response and Lipid Perioxidation in Volume Overloaded Rats
Source: Front Pharmacol. 2022 Jun 30;13:873169. doi: 10.3389/fphar.2022.873169 (PMC9280420; doi:10.3389/fphar.2022.873169)

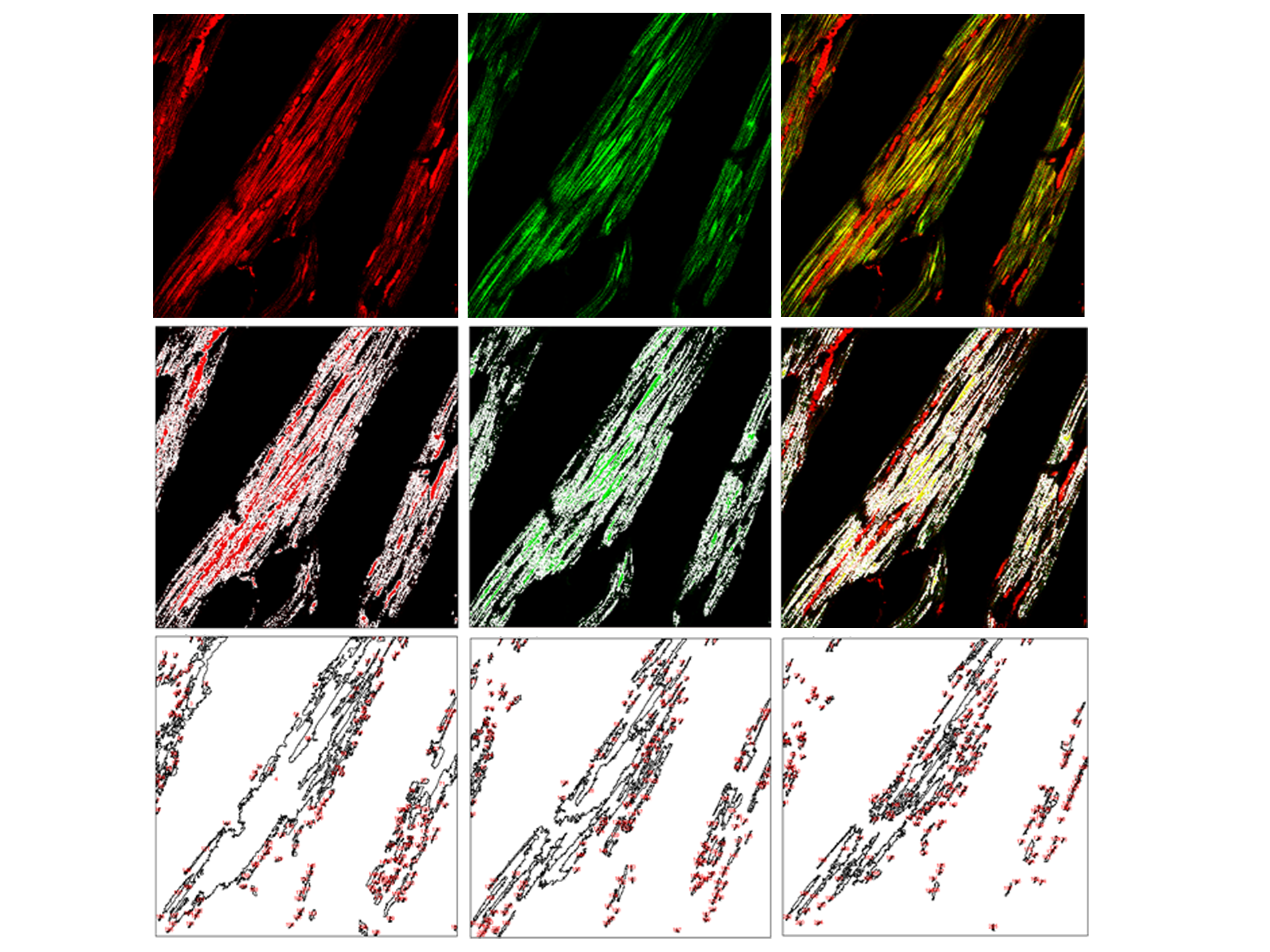

Supplement: Supplementary file 1 [file Image1.TIF]
